# Investigation of Hydrodynamic Behavior of Alginate Aerogel Particles in a Laboratory Scale Wurster Fluidized Bed

**DOI:** 10.3390/molecules24162915

**Published:** 2019-08-11

**Authors:** Işık Sena Akgün, Can Erkey

**Affiliations:** Department of Chemical and Biological Engineering, Koç University, Sarıyer, Istanbul 34450, Turkey

**Keywords:** alginate, aerogel, particles, fluidization, Wurster fluidized bed, hydrodynamics

## Abstract

The effects of design and operating parameters on the superficial velocity at the onset of circulatory motion and the residence time of alginate aerogel particles in a laboratory scale Wurster fluidized bed were investigated. Several sets of experiments were conducted by varying Wurster tube diameter, Wurster tube length, batch volume and partition gap height. The superficial velocities for Wurster tube with 10 cm diameter were lower than the tube with 8 cm diameter. Superficial velocities increased with increasing batch volume and partition gap height. Moreover, increasing batch volume and partition gap height led to a decrease in the particle residence time in the Wurster tube. The results showed that there is an upper limit for each parameter in order to obtain a circulatory motion of the particles. It was found that the partition gap height should be 2 cm for proper particle circulation. Maximum batch volume for the tube with 10 cm diameter was found as 500 mL whereas maximum batch volume was 250 mL for the tube with 8 cm diameter. The fluidization behavior of the aerogel particles investigated in this study could be described by the general fluidization diagrams in the literature.

## 1. Introduction 

Aerogels which were first synthesized in 1931 by Samuel Kistler are nanoporous materials with open pore structures [1]. Due to their high specific surface areas, low densities and high pore volumes, aerogels in the form of particles are attracting increasing attention for a wide variety of applications such as cosmetics, catalysis, drug delivery and adsorption [2,3,4,5]. Enhancements of some of the properties of aerogels such as mechanical strength, thermal stability and appearance by coating the surface of aerogel particles with a thin polymer layer may significantly improve the performance of aerogel-based products. 

There have already been several investigations to synthesize coated aerogel particles. Ulker et al. synthesized paracetamol loaded silica aerogel beads coated with an alginate aerogel layer by the dip coating method [6]. Silica alcogel core was immersed into a sodium alginate solution to trigger alginate layer formation which was then converted to an aerogel by drying with supercritical CO_2_. Cremaes et al. utilized the compressed CO_2_ antisolvent approach for coating silica aerogel with Eudragit RL 100 [7]. Recently, Alnaief et al. used a spouted bed to form multiple Eudragit L layers on silica aerogel microspheres [8]. They observed that Eudragit L solution droplets penetrated the pores of the aerogel particles in a short time. Moreover, high shrinkage of aerogel particles during coating occurred. To preserve the textural properties of the aerogels, PEG was successfully used as protection layer. Halim et al. coated the silica aerogel surface with a PVA film using a bottom spray-Wurster fluidized bed [9]. Coated aerogel particles had a higher compressive strength and thermal stability than uncoated particles.

Among these methods, coating in a Wurster fluidized bed is particularly suitable for small particles due to its smooth and continuous tube above the perforated plate which enables circulatory particle motion [10]. It was developed by Dr. Dale Wurster in the late 1950′s for tablet coating in the pharmaceutical industry [11]. There are four different zones in a Wurster fluidized bed which are tube, expansion, annular and transporting zone. In all Wurster fluidized beds, particle movement is achieved by pressure difference between annular and tube zone through the partition gap which is the opening between the annular zone and the Wurster tube. Particles are transported upward through the Wurster tube and then they decelerate into the expansion chamber and fall towards the annular zone with gravity. This path followed by particles in the bed is called as U-shaped trajectory of particles [12,13]. Particle fluidization behavior inside the bed depends on design parameters of the bed such as configuration of the perforated plate, Wurster tube size, partition gap height and also on operating parameters such air velocity and physical characteristic of particles such as porosity, density, shape and size [11].

Experimental investigations on hydrodynamic behavior of a wide variety of particles in Wurster fluidized beds were carried out. Che et al. developed two types of electrical capacitance tomography (ECT) sensors to evaluate gas-solid flow characteristics of sucrose pellets in the bed [14]. The measurements revealed that the partition gap height, superficial velocity and batch volume governed the particle fluidization regimes in the annular zone (minimum fluidization or bubbling phase) and the tube zone (core or dispersed flow). Guignon et al. compared hydrodynamic behavior of several types of particles which were semolina wheat, glass, alumina, polystyrene and PMMA [15]. Appropriate fluidization air flow rate was determined for circulatory motion of each particle type at a fixed partition gap height. They observed that air velocity in the Wurster tube should be at least twice as high as the particle terminal velocity for circulatory motion. In the work by Li et al., positron emission particle tracking experiments (PEPT) were performed using microcrystalline cellulose (MCC) spheres to determine effects of batch volume, partition gap height and the tube length on the cycle times and residence time distribution in different regions of the bed [13]. The particle residence time in the bed decreased by increasing the batch volume and decreasing the tube length. To the best of our knowledge, there are no studies in the literature on the hydrodynamic behavior of aerogel particles in a Wurster fluidized bed. 

An understanding of the hydrodynamic behavior of aerogel particles in a Wurster fluidized bed is necessary to provide insight into the relation between fluidized bed design parameters and particle fluidization behavior. Such data are also useful for design and scale-up of Wurster fluidized beds for a wide variety of treatment processes for aerogels such as impregnation and coating [11]. In this study, we investigated the hydrodynamic behavior of alginate aerogel particles in a self-made laboratory scale plexiglass Wurster fluidized bed. Several sets of experiments were conducted by varying Wurster tube diameter, Wurster tube length, batch volume and partition gap height. Superficial velocities for the onset of circulatory motion were measured as a function of batch volume and partition gap height. Upper working limits of each parameter for regular particle fluidization was determined. Particle residence times inside the tube were also measured for corresponding batch volumes, tube lengths and partition gap heights. 

## 2. Materials and Methods

### 2.1. Materials

Sodium salt of alginic acid from brown algae (low viscosity) and calcium chloride (anhydrous granular, >93%) were purchased from Sigma-Aldrich^®^ (Istanbul, Turkey). Ethanol (99.9% purity) was obtained from Isolab. 

### 2.2. Synthesis of Spherical Calcium Alginate Aerogel Beads

Spherical calcium alginate aerogel beads were prepared by CaCl_2_ induced gelation followed by solvent exchange with ethanol and supercritical drying with CO_2_. Details of the method are described elsewhere [16]. Briefly, 3 wt% aqueous solution of sodium alginate was added dropwise into a 0.2 M CaCl_2_ aqueous solution with constant stirring at 250 rpm. Ca^2+^ is usually used to trigger the gelation of the alginate [17]. Approximately, 11,000 drops of alginate were added to the 300 mL CaCl_2_ solution to obtain 500 mL aerogel. Alginate to CaCl_2_ ratio was 1.35:1 (*w*/*w*) on a dry basis. Spontaneously formed hydrogel beads were then converted to alcogels by stepwise solvent exchange of water with mixtures of ethanol and water (10%, 30%, 50%, 70%, 90% and lastly in 100% ethanol by volume) to prevent shrinkage of beads. Calcium alginate alcogels were then supercritically dried in an Applied Separations Speed SFE unit. First, the alcogel particles and a certain amount of ethanol were put in a 500 mL tubular vessel (length = 225 mm, internal diameter = 71 mm). The vessel was then placed in the oven. The inlet and outlet lines were connected to the vessel and the vessel was heated to 40 °C and pressurized with CO_2_ to 125 bars. Subsequently, outlet valve was opened and adjusted to start the extraction. After 8 h, the inlet valve was closed, and the vessel was depressurized slowly and spherical alginate aerogel particles were obtained. 

**Figure 1:**
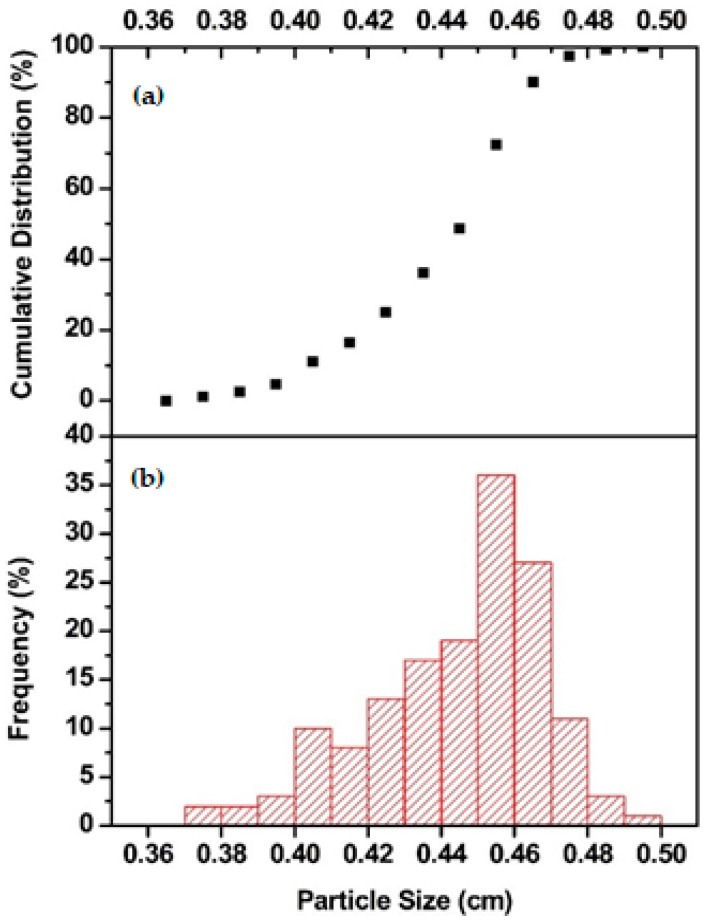
Particle size distribution of alginate aerogel particles in 500 mL batch volume before the experiments. (**a**) The cumulative particle size distribution. (**b**) The particle size distribution.

The Feret diameter (Calliper diameter) of 250 spherical alginate aerogel particles was measured by a Vernier calliper before and after each run [18]. Figure 1 shows the particle size distribution (PSD) of the aerogel particles in 500 mL batch volume before the experiments. The mean particle size was 4.40 ± 0.23 mm. The mass of the 250 spherical alginate aerogel particles was also measured with a balance (^©^Mettler Toledo TR, Istanbul, Turkey). The density of each particle was found by diving the particle mass by the particle volume. The mean density of spherical alginate aerogel particles was found as 0.045 ± 0.009 g/cm^3^.

### 2.3. Experimental Setup 

**Figure 2:**
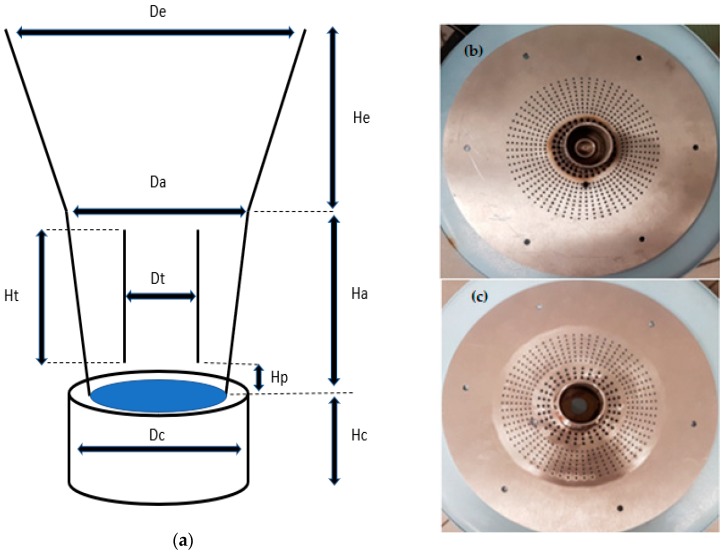
Schematic diagram of Wurster fluidized bed; (**a**) plexiglass expansion and annular chambers and stainless steel cylindrical chamber; (**b**) perforated plate design for narrow Wurster tube (diameter: 8 cm), (**c**) perforated plate design for wide Wurster tube (diameter: 10 cm), larger holes (diameter: 3.5 mm) under the tube zone and smaller holes (diameter: 2 mm) under the annular zone.

Hydrodynamic experiments were performed in a self-made laboratory scale batch type Wurster fluidized bed which is shown in Figure 2. The geometry of the components of the Wurster fluidized bed is summarized in Table 1. The upper part of the equipment consisted of an annular chamber, a conical expansion chamber and a cylindrical Wurster tube which were made from plexiglass in order to visually observe the particle flow. Annular and expansion chambers were welded to each other together. The bottom part which was made from stainless steel consisted of a cylindrical chamber and a perforated plate. The perforated plate was placed between the annular chamber and the cylindrical chamber. Two different perforated plates with holes with different diameters were used (Figure 2b,c) for two different cylindrical Wurster tubes with diameters of 8 cm (narrow) and 10 cm (wide). Generally, the diameter of Wurster tube is typically one half of the diameter of the perforated plate which was the case for the tube with 10 cm diameter [19]. The perforated plate had larger holes (diameter: 3.5 mm) in the section under the Wurster tube whereas the holes under the annular zone were smaller (diameter: 2 mm). 

The annular chamber, perforated plate and cylindrical chamber were connected via a flange. The Wurster tube was vertically placed above the perforated plate by a stainless-steel rod which was mounted on the perforated plate. Partition gap height between the perforated plate and the Wurster tube was changed by varying the rod height. 

For each run, particles were loaded from top of the expansion chamber and placed on the perforated plate. The fluidization behavior of particles was visually observed and also recorded with a camera (Samsung ISOCELL S5K2L1, Samsung Group, Istanbul, Turkey). Fluidization air entered the bed from the stainless-steel cylindrical chamber. Compressed air was delivered to the bottom of the chamber using the Atlas Copco GX4FF compressor (max pressure: 10 bars). A pressure gauge (MeI, 0–16 bar, Manometría e Instrumentación, S.l., Istanbul, Turkey) and a rotameter (LZM-15G, Changzhou Voha Instrument Co., Ltd, Istanbul, Turkey) were installed in the line at the outlet of the compressor to monitor the exit air pressure and flowrate. Measured volumetric flow rates and pressures of the fluidization air at the outlet of the compressor at steady state were used to calculate the air velocities. All velocities were calculated with Equation (1) which shows that mass flow rate of the fluidization air at the outlet of the compressor is equal to the mass flow rate of fluidization air in the Wurster bed and is given by:

(1) $$Q_{1}\rho_{1}=uA_{2}\rho_{2}$$

where Q_1_ is the measured volumetric flow rate at the outlet of the compressor, ρ_1_ is the corresponding density of air at measured pressure, A_2_ is the area of perforated plate, ρ_2_ is the corresponding density of air at atmospheric pressure and *u* is the superficial air velocity in the bed. 

## 3. Results and Discussion

### 3.1. General Particle Movement

**Figure 3:**
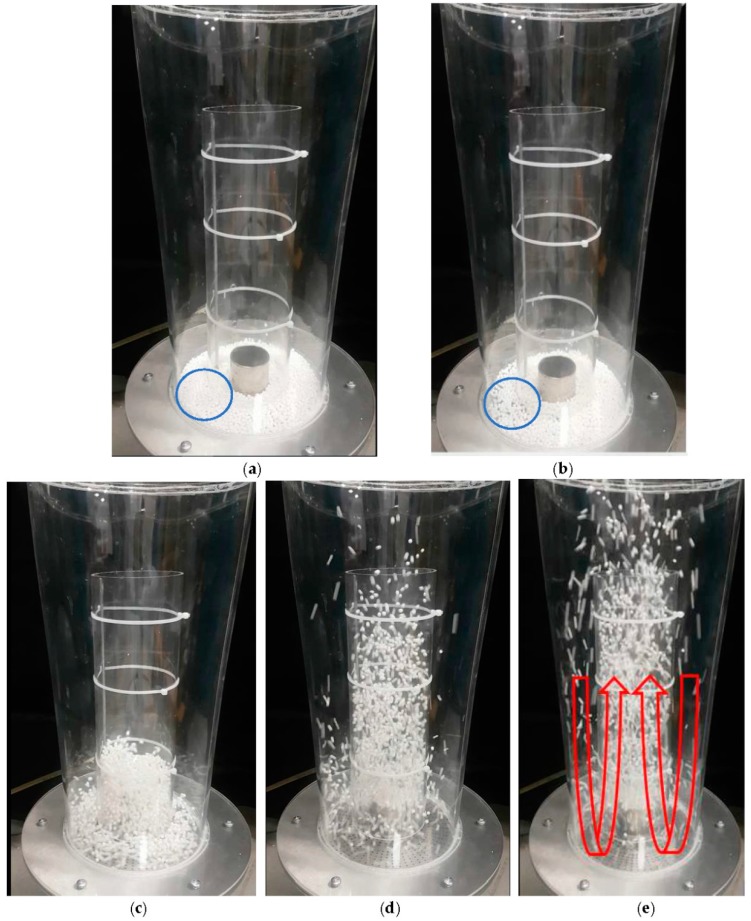
Regimes of fluidization of alginate aerogel particles in the Wurster fluidized bed with 150 mL batch volume, 2 cm partition gap height and the wide tube. (**a**) There was no air flow inside the bed. (**b**) Minimum fluidization (the minimum fluidization air velocity: 0.19 ± 0.01 m/s). Blue circled area represents particle behavior at minimum fluidization velocity. They started vibrating and bed porosity increased in comparison to same blue circled area in (**a**). (**c**) Bubbling phase. (**d**) Turbulent flow. Lean and dispersed particle movement at the outlet of the tube. (**e**) The U-shaped trajectory of particles with a circulatory motion (superficial velocity: 2.4 ± 0.1 m/s).

For aerogel particles, different regimes of fluidization in a Wurster fluidized bed were observed which depended on the superficial air velocity. As an example, the movement of the particles in the Wurster fluidized bed with 150 mL batch volume, 2 cm partition gap and wide tube (Run #9) is shown in Figure 3. 

At the beginning of each run (Table 2), particles were placed uniformly on the perforated plate (Figure 3a). Compressed air was then supplied to the bed at a particular flow rate. Particles started to vibrate when a certain flow rate was reached which is indicative of a transition from a fixed bed to a fluidized bed (Figure 3b). At the onset of fluidization, the weight of particles equals drag force exerted by the ascending gas. At that point porosity of bed increases and particles exhibit ‘liquidlike’ behavior. Corresponding air velocity at that point is minimum fluidization velocity, (*u_mf_*) [20]. After reaching the *u_mf_*, air flow rate was slightly increased, and particles moved towards to the Wurster tube by the pressure difference between annular zone and tube zone due to the Venturi effect. At that step, air velocity was not sufficient for particle elevation inside the Wurster tube so that particles fluidized at bubbling phase (Figure 3c). By further increasing the air flow rate, turbulent flow of the particles was achieved. In this step, particles reached the upper edge of the tube in dilute and disperse phase and then fell into the annular zone (Figure 3d). Particles started following the U-shaped trajectory and fully circulated inside the bed when a particular air velocity was reached (Figure 3e). 

### 3.2. Effects of Design and Process Parameters on Particle Fluidization Behavior 

The conditions for the various runs which were conducted are given in Table 2. At the beginning of each run the *u_mf_* was measured at the vibration point of the particles. All measurements were repeated three times and the *u_mf_* was found nearly identical for each run. The average of the 40 runs is 0.19 ± 0.01 m/s. Thus, one can say that that the *u_mf_* does not depend on the design parameters of the bed or operating parameters such as batch volume. It just depends on the physical properties of the particle. *u_mf_* can also be calculated using an expression which is obtained by rearranging Ergun Equation [21] and is given by:

(2) $$\left(\rho_{p}-\rho_{f}\right)g=150\frac{\mu_{f}\;V_{mf}\;\left(1-\varepsilon_{mf}\right)}{\varphi_{s}^{2}D_{p}^{2}\varepsilon_{mf}^{3}}+1.75\frac{\rho_{f}V_{mf}^{2}}{\varphi_{s}\;D_{p}\;\varepsilon_{mf}^{3}}$$

Parameters of Ergun Equation are given in Table 3. Void fraction under minimum fluidization ($\varepsilon_{mf}$) was calculated with Wen & Yu correlation [22]. Density and viscosity of air were taken from literature [23]. Particle sphericity was assumed as 1 for spherical aerogel particles. *u_mf_* was calculated as 0.18 m/s using Equation (2). Thus, Ergun Equation (2) gave a good estimate of measured *u_mf_* of aerogel particles. 

**Figure 4:**
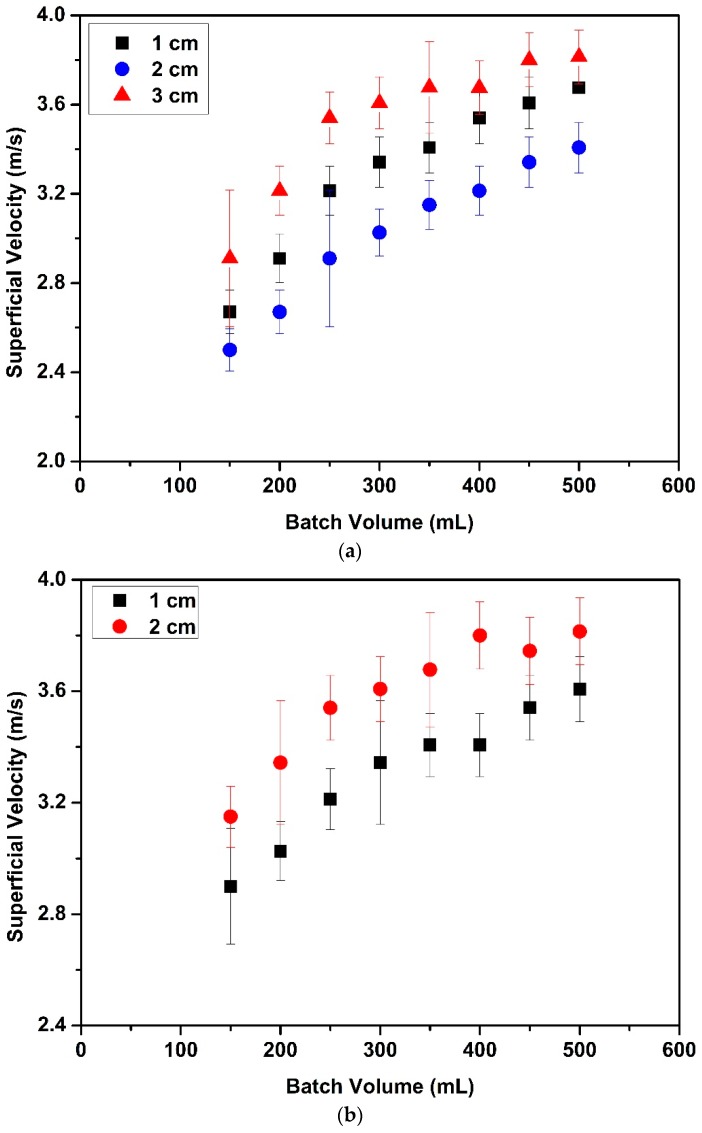
Superficial velocities at onset of circulatory motion in the bed for different batch volumes and partition gap heights: (**a**) The wide Wurster tube (diameter: 10 cm); (**b**) the narrow Wurster tube (diameter: 8 cm).

Superficial velocities were also measured at onset of circulatory motion. Effects of partition gap height, tube diameter and batch volume on the superficial velocities are given in Figure 4. Figure 4a,b illustrate that the superficial velocities for the narrow tube were higher than for the wide tube. For instance, the superficial velocity for 150 mL batch volume with 2 cm partition gap height (Run #33) with the narrow tube was 1.3 times higher than in the wide tube with same partition gap height (Run #9). This can be attributed to the fact that a higher suction pressure was needed to move particles from the annular zone to the tube zone for the narrow tube since the particles traverse a larger distance in the annual zone for the narrow tube. Thus, the superficial velocity is higher to provide the required suction pressure for fluidization. It was also found that variations of the process (batch volume) and the design (partition gap height and tube length) parameters resulted in more significant changes in the fluidization behavior of aerogel particles for the narrow Wurster tube than the wide Wurster tube. 

Figure 4a,b also show the effect of batch volume on superficial velocity. Superficial velocity linearly increased when the batch volume increased from 150 mL to 250 mL. Increasing superficial velocity with increasing batch volume was expected since a higher flow rate of air is required to carry more particles to a certain height. There was a change in the rate of increase when the batch volume was increased to 250 mL from 500 mL. This change can be attributed to the hydrostatic pressure effect which increases the mass flow rate of particles from the annular zone to the tube zone. This additional force results in a lower superficial velocity than expected. A similar phenomenon was also observed by Chan et al. for fluidization of sugar pellets in Wurster fluidized bed [24]. 

Experiments with batch volumes larger than 500 mL were also carried out. It was observed that some of the particles were fluidized in the annular zone which can be attributed to the Venturi effect which is mainly responsible for particle movement from the annular zone to the tube zone [25]. Air enters the bed through the holes of perforated plate. At that point, air velocity has only a vertical component. Venturi effect is observed when the air passes through the partition gap. The Wurster tube causes a pressure difference between the annular zone and the tube zone leading to a suction pressure at the bottom of the partition gap. Thus, air velocity obtains a horizontal component which decreases the magnitude of the vertical component of the air velocity at constant air flow rate. Particles move from annular zone to the tube zone because of the horizontal component of the air velocity [11]. Inside the Wurster tube, vertical component of the air velocity should be sufficiently higher than the horizontal component for particle elevation. However, high batch volumes above 500 mL lead to a decrease in the pressure difference between the annular zone and the Wurster tube zone resulting in a decrease of the vertical component of the velocity. Thus, one could see random particle fluidization in these runs. Some of the particles recirculated inside the tube and some of the particles accumulated at the bottom edge of the Wurster tube. Some of the particles were stationary inside the Wurster tube zone. Moreover, some of the particles were observed to stick to each other and agglomerated in various zones in the bed. This is because the particles became electrostatically charged due to collisions with other particles and the wall of the bed because of high velocities. 

**Figure 5:**
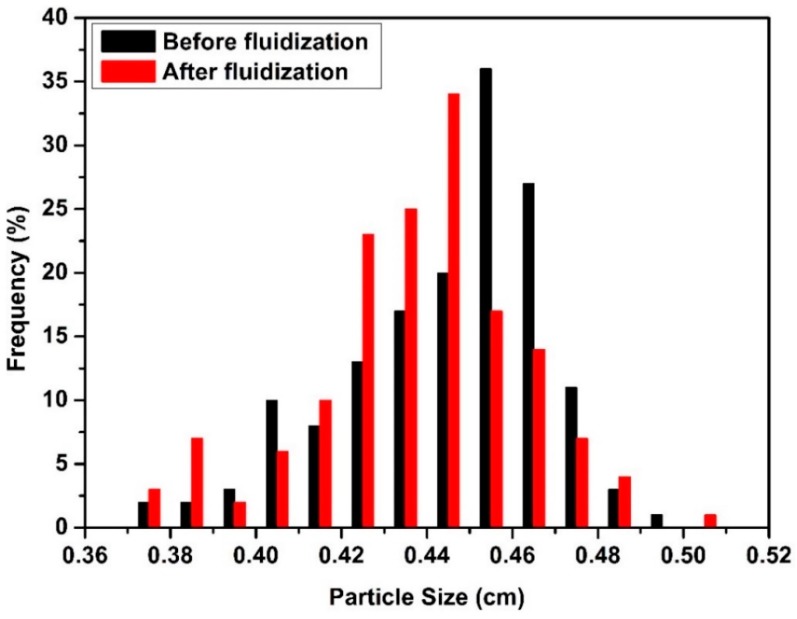
Particle size distribution of aerogel particles before and after Run #16.

**Figure 6:**
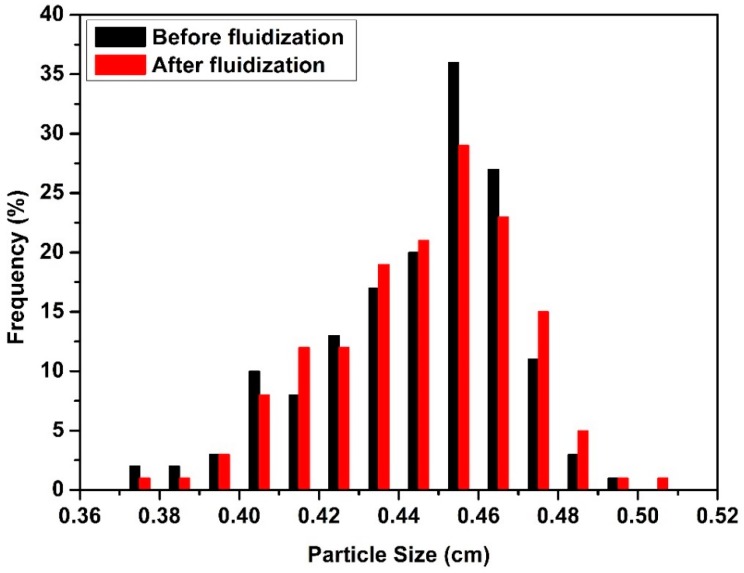
Particle size distribution of aerogel particles before and after Run #11.

Such a high superficial velocity also crushed some of the aerogel particles at higher batch volumes. Figure 5 shows that PSD of 500 mL batch volume before and after Run #16. One can see that the size of particles decreased after the fluidization and PSD shifted to the left. Crushed particles destroyed regular particle fluidization due to their different fluidization pathway. Crushed particles stuck to bed and tube walls which resulted in an increase in the number of agglomerates. There was no significant change in PSD with batch volumes lower than 500 mL. Figure 6 illustrates PSD of 250 mL batch volume before and after Run #11. Thus, a batch of volume of 500 mL can be expected to be the upper limit for operation for the tube with the 10 cm diameter. The same behavior was observed at a much smaller batch volume (250 mL compared to 500 mL) for the narrow tube due to lower ratio of tube diameter/perforated plate diameter which resulted in higher velocity for smaller batch volumes.

The effects of changing the partition gap height were also investigated. When partition gap height was increased from 1 cm to 2 cm with the narrow tube (Figure 4b), it was found that superficial velocities increased. Since pressure difference between the annular zone and the Wurster tube zone decreases with increasing partition gap height, the vertical component of the velocity inside the tube decreases [26]. Further increasing partition gap height from 2 cm to 3 cm induced a transition from regular fluidization to bubbling phase which resulted in over fluidization in the annular zone. Therefore, superficial velocities were not measured for 3 cm gap height. 

It is interesting to note that superficial velocities did not increase with increase of partition gap height from 1 cm to 2 cm for the wide tube (Figure 4a). This phenomenon may be explained by the following factors. With 1 cm partition gap, particles accumulated at the lower edge of the tube, stacked there and formed clusters before moving into the tube due to higher ratio of particle size/partition gap height. After a certain time, these clusters disrupted circulatory motion of the particles. Therefore, superficial velocity at onset of circulatory motion increased with 1 cm partition gap height for both dissociating clusters into individual particles and achieving circulatory motion. Increasing the partition gap height from 1 cm to 2 cm provides a larger opening to the particles at above the partition gap. Particles followed circulatory motion at lower superficial velocities with 2 cm partition gap height than with 1 cm partition gap height since larger partition gap height prevented the formation of agglomerates. Subsequently, increasing partition gap height from 2 cm to 3 cm, significantly increased superficial velocities. Some of the aerogel particles were crushed and agglomerated at these higher velocities and disrupted the circulatory motion. Consequently, partition gap height should not exceed 2 cm for both tubes.

Finally, we developed three correlations to predict the superficial velocity at onset of circulatory motion as a function of batch volume and tube diameter. It was not possible to represent superficial velocities with one equation since velocity for the 10 cm tube first increased and then decreased with partition gap height. Therefore, we separately developed three correlations for each partition gap height. The correlation for 1 cm and 2 cm partition gap heights is given by:

(3) $$u=\alpha {\left(TD\right)}^{\beta }{\left(BV\right)}^{\gamma }$$

where *u* is the superficial velocity at onset of circulatory motion, *TD* is the tube diameter and *BV* is the batch volume.

The constants *α*, *β* and *γ* in the correlation were determined for 1 cm and 2 cm partition gap heights, separately. A nonlinear regression solver in PYTHON was used to find the constants by fitting the model results to experimental data. For 3 cm partition gap height, the correlation was developed as a function of batch volume and is given by:

(4) $$u=\delta {\left(BV\right)}^{\theta }$$

**Figure 7:**
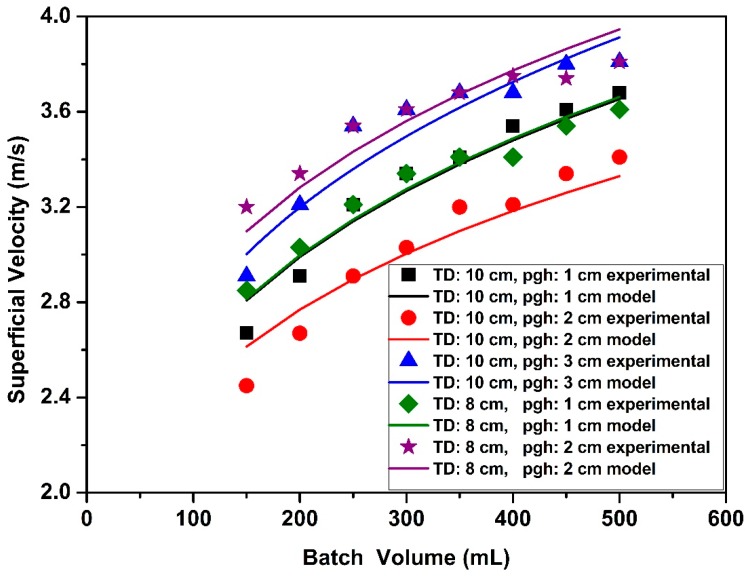
Comparison of experimental data with model predictions for superficial velocity at onset of circulatory motion in the bed. (TD: Wurster tube diameter, pgh: partition gap height).

The constants *α*, *β* and *γ*, δ and θ in the correlations and calculated $R^{2}$ values are given in Table 4. Figure 7 shows the comparison of experimental data and model results. There is a good agreement between the model results and experimental data.

### 3.3. Fluidization Regimes of Alginate Aerogel Particles in the Classical and Modified Geldart Classification

**Figure 8:**
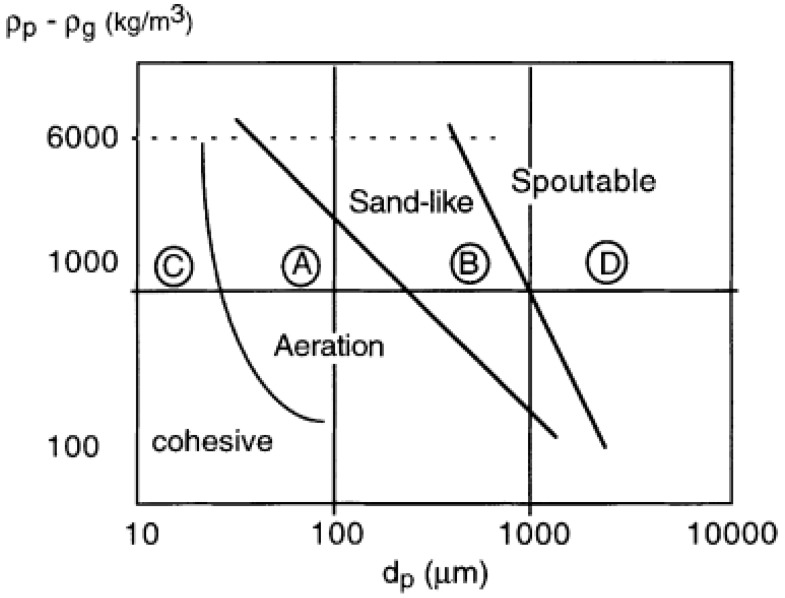
The Geldart classification of particles [28].

In the literature, particle fluidization behavior in fluidized beds generally falls into one of the four regions as classified by Geldart [27]. These regions (A, B, C, D) are shown in Figure 8 and depend on particle size and density difference between the solids and the fluid at ambient temperature and the pressure [28]. In Geldart A region, particles fluidize well. Most particles investigated in fluidization studies belong to this region. In Geldart B region, bubbling fluidization regime is observed and the fluidization behavior of these particles is highly affected by the gas bubbles [29]. In Geldart C region, agglomeration and bubbling fluidization is observed and it is often difficult to fluidize the particles [29,30]. In Geldart D region, high fluidization velocities are needed due to large particle sizes and high densities [29]. The aerogels used in this study have very low density ($\rho_{p\;}$-$\;\rho_{g}:$ 43.8 kg/m^3^) and large particle size ($d_{p}:$ 4400 µm). Therefore, they fall outside these regions. At low superficial velocities, aerogel particles may fall into region B due to bubble formation. At moderate superficial velocities and small batch volumes, aerogel particles fluidized well and may fall into region A. At higher superficial velocities and batch volumes, agglomerates were observed as in region C.

**Figure 9:**
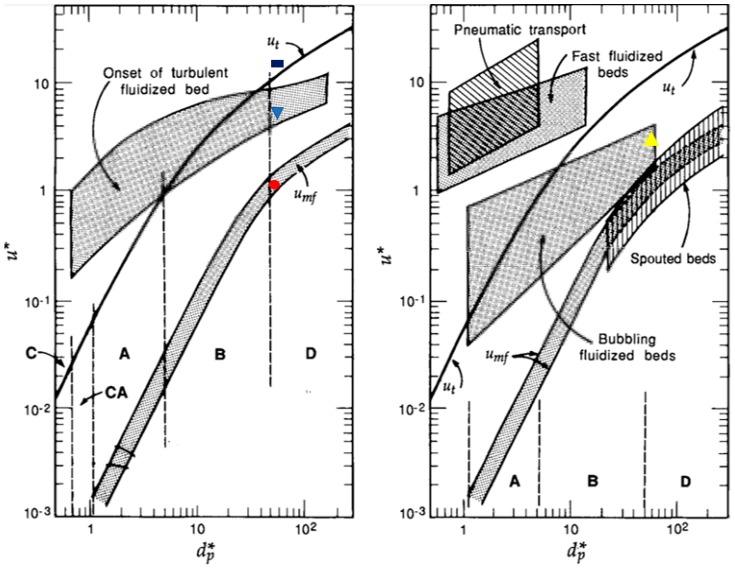
General fluidization regime diagrams from packed bed to pneumatic transport of particles [31]. Letters A, B, C, and D refer to the Geldart classification of particles. *u*_t_ is the terminal velocity which is free-fall velocity of a particle through the fluidization gas.

Thus, classical Geldart classification is not quite suitable to describe the fluidization behavior of alginate aerogel particles. Superficial velocity also affects the particle fluidization behavior as discussed above. Figure 9 shows two diagrams which combine most of the information about different fluidization regimes based on air velocity and particle diameter [31]. These diagrams also include modified boundaries of Geldart classification. 

In Figure 9, the minimum fluidization regime occurs only for Geldart A, B and D particles. Bubbling regime occurs for Geldart A and B particles for a wide range of particle size and air velocity whereas Geldart D particles can be bubbled in a very narrow region of particles size. Onset of the turbulent fluidized bed regime is above the terminal velocity for Geldart C and A particles whereas it is below the terminal velocity for Geldart D particles. Besides the minimum, bubbling and turbulent fluidization regimes discussed above, three other regimes are given in Figure 9 which are pneumatic transport, fast fluidized and spouted bed. These three regimes were not observed for the aerogel particles with a diameter of 4.40 mm in our Wurster fluidized bed.

The x and y-axes of Figure 9 are dimensionless diameter, d_p_*, and dimensionless velocity, u* which can be calculated by Equations (5) and (6) given by:

(5) $$d_{p}^{*}=d_{p}{\left\lceil \frac{\rho_{g}\left(\rho_{p}-\;\rho_{g}\right)g}{\mu^{2}}\right\rceil }^{1/3}$$

(6) $$u^{*}=u{\left\lceil \frac{\rho_{g}^{2}}{\mu \left(\rho_{p}-\;\rho_{g}\right)g}\right\rceil }^{1/3}$$

where $d_{p}$ is the particle diameter, $\rho_{p}$ is the particle density, $\rho_{g}$ is the gas density, μ is the gas viscosity, and u is the superficial velocity. Superficial velocities were also measured at the onset of fluidization, bubbling, turbulent flow regimes and circulatory motion for Run #9 to see if these diagrams also captured the fluidization behavior of aerogel particles. d_p_* and u* were calculated using superficial velocity and particle diameter for Run #9. The velocities, dimensionless velocities, diameter and dimensionless particle diameter are given in Table 5. 

The four points in Table 5 are also plotted in Figure 9 as red solid circle, blue triangle, yellow triangle and black rectangle. One can see that the dimensionless particle diameter is in Region D for the aerogel used in this study. The red circle representing the data obtained for the onset of fluidization for Run #9 falls right inside the area representing minimum fluidization region in Figure 9. The yellow triangle representing the data obtained at the onset of bubbling regime also falls inside the area covering bubbling region in Figure 9. The blue triangle representing the data obtained for the onset of turbulent flow regime in Run #9 also falls in the area representing the onset of turbulent flow region. These indicate that Figure 9 also captures the fluidization behavior of aerogel particles investigated in this study. There is no region for circulatory motion in Figure 9. The black rectangle representing the data obtained for the onset of circulatory motion just falls above the line for terminal velocity. 

### 3.4. Particle Residence Time in the Wurster Tube 

**Figure 10:**
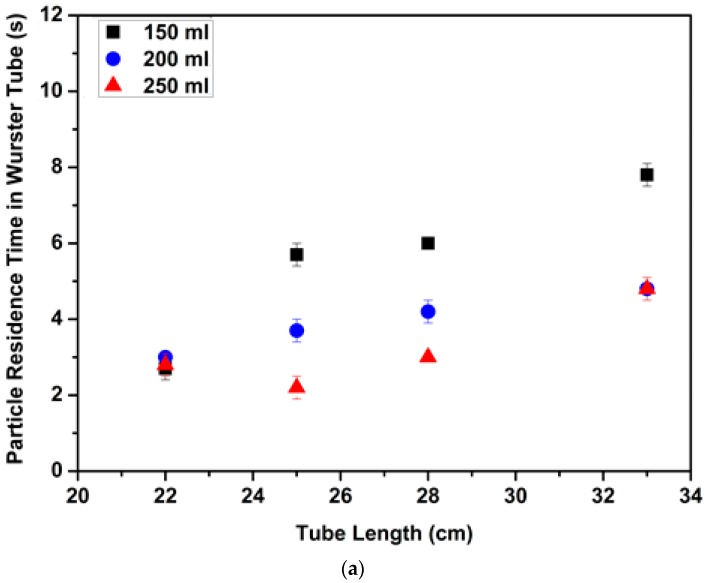

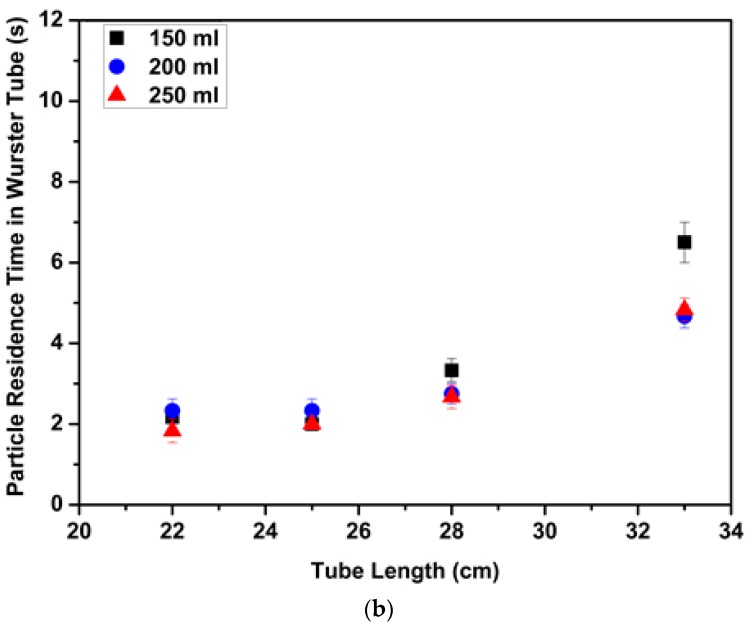
Variation of aerogel particle residence time in the narrow Wurster tube with batch volume, tube length for (**a**) 1 cm partition gap height; (**b**) 2 cm partition gap height.

Particle residence time in the Wurster tube is another significant parameter. In this study, an aerogel particle which had a diameter of 4.40 mm and a density of 0.045 g/cm^3^ was selected and dyed with green coloring. At the beginning of each run, the same green particle was placed near the bed wall. Each run was operated at a constant velocity (3.7 m/s) which was higher than superficial velocities at onset of circulatory motion of each run. The particle movement in the bed was monitored with a camera (Samsung ISOCELL S5K2L1) during the run which lasted 15 s. Particle residence time in the tube was measured by timing the entrance and exit of the green colored particle to and from the narrow Wurster tube with lengths of 22 cm, 25 cm, 28 cm and 33 cm. Exclusively, batch volumes from 150 mL to 250 mL were investigated with 1 cm and 2 cm partition gap heights since fluidization behavior of the aerogel particles for the narrow tube which was 33 cm long started to change for batch volumes above 250 mL. After each run PSD was measured and there was no significant change in PSD. Effects of tube length, batch volume and partition gap height on particle residence time in the Wurster tube are shown in Figure 10. 

Figure 10a,b show the effect of batch volume on mean values of particle residence time in the tube. Increasing batch volume resulted in a decrease in particle residence time. On one hand, as explained in Section 3.2. the horizontal component of the air velocity increased with increasing batch volume leading to a decrease in the vertical component of the air velocity in the tube. On the other hand, number of particles in the tube zone, which is one of the driving forces for particle elevation inside the Wurster tube, increased with increasing horizontal component of the air velocity resulting in shorter particle residence time in the tube. 

Figure 10a,b also illustrate the effect of tube length on the particle residence time. The most pronounced effect of increasing the tube length is the increase in particle residence time due to a longer path inside the tube. Moreover, increasing tube length had different effects when batch volume was varied. The highest change in particle residence time with increasing tube length was observed for 150 mL batch volume. For example, a 50% increase in tube length resulted in a 200% increase in residence time (Figure 10a). Particle residence time with 250 mL batch volume slightly increased when tube length increased from 22 cm to 33 cm due to the higher number of particles in the annular zone (Figure 10a,b). 

Figure 10a,b also show how particle residence time changes with partition gap height. Particle residence time decreased when partition gap height increased from 1 cm to 2 cm due to increasing horizontal component of the air velocity as explained Section 3.2. Increasing horizontal component of the air velocity with increasing partition gap height resulted in a higher number of particles at the tube zone which contributed to particle elevation inside the tube. Li et al. investigated the effect of partition gap height on the cycle time of microcrystalline cellulose (MCC) pellets [13]. Cycle time increased when partition gap height increased from 1 cm to 1.5 cm. Subsequently, increasing partition gap height from 1.5 cm to 2 cm resulted in decreasing cycle time. However, we did not observe an increase in residence time with increasing partition gap height from 1 cm to 1.5 cm or from 2 cm to 2.5 cm most likely due to the different fluidization properties of aerogel and MCC particles. 

Particle residence time with 150 mL batch volume significantly changed with partition gap height for tubes with lengths of 25 cm, 28 cm and 33 cm (Figure 10a,b). Residence times decreased 2–3 s on average when the partition gap height increased from 1 cm to 2 cm for 150 mL batch volume. There was no significant change in the particle residence times with partition gap height for batch volumes of 200 mL and 250 mL (except 33 cm). Thus, one can say that design parameters control the particle residence time for the smallest batch volume used in the experiments.

Residence time of the particles with diameters of 4.60 mm and 4.20 mm were also investigated for 22 cm length and batch volumes of 150 mL and 250 mL with 1 cm partition gap height. These particles were dyed with green coloring. Same measurement method used for the particle with the diameter of 4.40 mm was used to measure residence time of the small and large particles. It was found that the residence time of the large and the small particles in the narrow Wurster tube were almost the same (Table 6). We expected that large particles will move up more slowly than small ones due to their higher weight. This surprisingly similar residence time of small and large particles was also recorded by Li et al. for large and small MCC particles in the Wurster tube [13]. This was attributed to the collisions between small and large particles. Velocity of small particles decreases whereas velocity of the large particles increases due to collisions. This gives similar residence time for all particles.

## 4. Conclusions

The hydrodynamic behavior of alginate aerogel particles in a laboratory scale Wurster fluidized bed was investigated for the first time. Effects of design and operating parameters on particle fluidization behavior and particle residence time in the Wurster tube were studied using different batch volumes (150–500 mL), at different partition gap heights (1–3 cm) and with different tube diameters (8, 10 cm). Increasing partition gap height, decreasing tube diameter and increasing batch volume resulted in an increase in superficial velocities for onset of circulatory motion. 500 mL and 250 mL batch volumes could be handled using tubes with 10 cm diameter and 8 cm diameter, respectively. Maximum partition gap height was determined as 2 cm for both of the tubes. 

Effect of physical properties of the aerogel particles on their fluidization behavior was discussed based on classical and modified Geldart classifications. It was found that aerogel particles fall outside the classical Geldart classification. Results also indicated that fluidization behavior of these aerogel particles could be described very well by the modified Geldart classification in the literature on fluidized beds. The data for aerogels fall right into the generalized regions for minimum fluidization, bubbling and onset of turbulence for a wide variety of particles. We also developed three correlations to predict the superficial velocity at onset of circulatory motion which may be helpful to generalize the results for different geometries and operating conditions of the Wurster bed.

Particle residence time in the Wurster tube was also measured using different batch volumes (150–250 mL), with different partition gap heights (1, 2 cm) and with different tube lengths (22–33 cm). Residence time increased with increasing tube length whereas particles spent shorter time in the tube with increasing partition gap height. It was found that the particle fluidization pattern was controlled by design parameters for smaller batch volume whereas process parameters dominated particle trajectory for larger batch volume. It was also found that particle diameter does not significantly affect the particle residence time in the Wurster tube. 

## Figures and Tables

**Table 1:** Dimensions of the components of the Wurster fluidized bed.

Variable	Value
Upper diameter of the expansion chamber, De (mm)	500
Upper diameter of the annular chamber, Da (mm)	230
External diameter of the cylindrical chamber, Dc (mm)	300
Internal diameter of the perforated plated, Dp (mm)	180
Internal diameter of Wurster tube, Dt (mm)	96, 76
Height of the expansion chamber, He (mm)	400
Height of the annular chamber, Ha (mm)	450
Height of the cylindrical chamber, Hc (mm)	300
Length of the Wurster tube, Ht (mm)	220, 250, 280, 330
Height of partition gap, Hp (cm)	1, 2, 3

**Table 2:** Design and process parameters for superficial velocity measurements.

Run	Partition Gap Height (cm)	Batch Volume (mL)	Tube Diameter ^1^ (cm)
1	1	150	10
2	1	200	10
3	1	250	10
4	1	300	10
5	1	350	10
6	1	400	10
7	1	450	10
8	1	500	10
9	2	150	10
10	2	200	10
11	2	250	10
12	2	300	10
13	2	350	10
14	2	400	10
15	2	450	10
16	2	500	10
17	3	150	10
18	3	200	10
19	3	250	10
20	3	300	10
21	3	350	10
22	3	400	10
23	3	450	10
24	3	500	10
25	1	150	8
26	1	200	8
27	1	250	8
28	1	300	8
29	1	350	8
30	1	400	8
31	1	450	8
32	1	500	8
33	2	150	8
34	2	200	8
35	2	250	8
36	2	300	8
37	2	350	8
38	2	400	8
39	2	450	8
40	2	500	8

^1^ Length of the narrow and the wide Wurster tube was 33 cm for each run.

**Table 3:** Parameters of Ergun equation.

Variable	Value
Particle density ($\rho_{\begin{array}{c} p \end{array}}$) (kg/m^3^)	45
Fluid density ($\rho_{f})$ (kg/m^3^)	1.1684 [23]
Viscosity of the fluid ($\mu_{f})$ (*kg/*(*m.s*))	0.00001825 [23]
Void fraction under minimum fluidization ($\varepsilon_{mf})$	0.415
Particle sphericity $\left(\varphi_{s}\right)$	1
Particle diameter ($d_{p})$ (mm)	4.40

**Table 4:** Summary of the model constants.

PGH (cm)	TD (cm)	α	β	γ	δ	θ	$R^{2}$
1	8	0.9632	−0.011	0.2186	-	-	0.943
1	10	0.9632	−0.011	0.2186	-	-	0.943
2	8	5.5160	−0.762	0.2011	-	-	0.953
2	10	5.5160	−0.762	0.2011	-	-	0.953
3	10	-	-	-	0.997	0.220	0.931

**Table 5:** The particle diameter, measured superficial velocities, calculated d_p_* and u* for Run #9.

Measured Superficial Velocity (*u*) (m/s)	Dimensionless Velocity $\left(u^{*}\right)$	Particle Diameter $\left(d_{p})\;*10^{3}\;(m\right)$	Dimensionless Particle Diameter $\left(d_{p}^{*}\right)$
At onset of fluidization: 0.19 ± 0.01	1.06	4.40 ± 0.23	50.46
At onset of bubbling regime: 0.53 ± 0.02	2.96	4.40 ± 0.23	50.46
At onset of turbulent flow regime: 0.97 ± 0.03	5.41	4.40 ± 0.23	50.46
At onset of U-shape trajectory: 2.4 ± 0.02	13.40	4.40 ± 0.23	50.46

**Table 6:** Particle residence times for 150 mL and 250 mL batch volumes and particle diameters with 4.20 mm, 4.40 mm and 4.60 mm, respectively.

	Particle Diameter (mm)		
Batch Volume (mL)	4.20	4.40	4.60
150	3.7 ± 0.6 s	3.8 ± 0.3 s	3.6 ± 0.9 s
250	3.0 ± 0.8 s	2.8 ± 0.3 s	3.0 ± 1.0 s
